# Supplementary material for: The Impact of the CYP2D6 and CYP1A2 Gene Polymorphisms on Response to Duloxetine in Patients with Major Depression
Source: Int J Mol Sci. 2023 Aug 30;24(17):13459. doi: 10.3390/ijms241713459 (PMC10487921; doi:10.3390/ijms241713459)
Supplement: Supplementary file 1 [file ijms-24-13459-s001.zip › ijms-2535642-supplementary.pdf]

Table S1. Detailed protocols for genotyping CYP2D6 and CYP1A2.

Polymorphism	Primers	Preliminary Denaturation	Amplification	Elongation	Restriction
CYP1A2 *1D	Cyp1A21DF 5′-TGA GCC ATG ATT GTG GCA TA-3 ′ CYP1A21DR 5′-AGG AGT CTT TAA DATA GGA CCC AG-3 ′	5 min, 94 ° C	35 cycles 30 s, 94 ° C 10 s, gradient 49 ° C 30 s, 72 ° C	5 min, 72 ° C	Ndei Eurx*
CYP1A2 *3	Cyp1A23F 5'-AGC CCT TGA GTG AGA AGA TG-3 ' Cyp1A23r 5'- GGT CTT GCT CTG TCA CTC A-3 '	5 min, 94 ° C	35 cycles 30 s, 94 ° C 10 s, gradient 58 ° C 30 s, 72 ° C	5 min, 72 ° C	Mluci New England Biolabs
CYP1A2 *1F	CYP1A2FF 5′-CAC CCT GCC AAT CTC AAG CAC-3 ′ CYP1A21FR 5′-AGA AGC TCT GTG GCC GAG AAG G-3 ′	3 min, 94 ° C	30 cycles 10 s, 98 ° C 30 s, 55 ° C 1 min, 72 ° C	5 min, 72 ° C	APAI New England Biolabs
CYP2D6 *4	Primer C: 5'-GCC TTC GCC AAC CAC TCC G-3 ' Primer D: 5'- AAA TCC TGC TCT TCC GAG GC-3 ′	1 min, 94 ° C	35 cycles 30 s, 94 ° C 30 s, 59 ° C 30 s, 72 ° C	5 min, 72 ° C	Wales, New England Biolabs
CYP2D6 *3	Primer E: 5'-gat gag ctg cta act gag ccc-3 ' Primer F: 5'-CCG AGA GCA TAC TCG GGA C-5 '	1 min, 94 ° C	35 cycles 30 s, 94 ° C 30 s, 59 ° C 30 s, 72 ° C	5 min, 72 ° C	Hpaii, Eurx

*Due to the difficulty of reading restrictive stripes on agarose gel the TAQman SNP set was also used in identifying the presence of the allel *1D Genotyping Assay C__60142977_10, Thermo Fischer Scientifi.

Table S2. Distribution of gene polymorphism variants and duloxetine metabolic scores.

CYP2D6						CYP1A2								
*4	DMS*	n (%)	*3	DMS	n (%)	*1D (-2464del T)	score	n (%)	*1F (-164A→C)	DMS	n (%)	*3 (1545T→C)	DMS	n (%)
AA	1	6 (6%)	*1/*1	3	100 (100%)	*1/*1	3	85 (85%)	AA	3	36 (36%)	TT	3	39 (39%)
GA	2	34 (34%)	*1/*3	2	0 (0%)	*1/*1D	4	13 (13%)	CA	4	53 (53%)	TC	2	53 (53%)
GG	3	60 (60%)	*3/*3	1	0 (0%)	*1D/*1 D	5	2 (2%)	CC	5	11 (11%)	CC	1	8 (8%)

Data expressed as n (%). *DMS - duloxetine metabolic score ranges from 1 to 5, which denotes the impact on duloxetine metabolism. This score allows for further determination of the duloxetine metabolic index separately for CYP2D6 and CYP1A2: -poor metabolizer - 1, intermediate metabolizer - 2, normal metabolizer - 3, rapid metabolizer - 4, and ultra-rapid metabolizer - 5.
